# Biomarkers in Glycogen Storage Diseases: An Update

**DOI:** 10.3390/ijms22094381

**Published:** 2021-04-22

**Authors:** Alberto Molares-Vila, Alberte Corbalán-Rivas, Miguel Carnero-Gregorio, José Luís González-Cespón, Carmen Rodríguez-Cerdeira

**Affiliations:** 1 Bioinformatics Platform, Health Research Institute in Santiago de Compostela (IDIS), SERGAS-USC, 15706 Santiago de Compostela, Spain; amolares@hotmail.com; 2 Local Office of Health Inspection, Health Ministry at Galician Autonomous Region, 27880 Burela, Spain; alberte.corbalan.rivas@sergas.es; 3 Department of Molecular Diagnosis (Arrays Division), Institute of Cellular and Molecular Studies (ICM), 27003 Lugo, Spain; miguel.carnero.gregorio@hotmail.com; 4 Efficiency, Quality, and Costs in Health Services Research Group (EFISALUD), Galicia Sur Health Research Institute (IIS Galicia Sur), SERGAS-UVIGO, 36213 Vigo, Spain; epi@uvigo.es; 5 Dermatology Department, Complexo Hospitalario Universitario de Vigo (CHUVI), Meixoeiro Hospital, SERGAS, 36213 Vigo, Spain

**Keywords:** Glycogen storage disease (GSD), Pompe disease, hepatic GSD, muscle GSD, biomarkers, Glycogen metabolism

## Abstract

Glycogen storage diseases (GSDs) are a group of 19 hereditary diseases caused by a lack of one or more enzymes involved in the synthesis or degradation of glycogen and are characterized by deposits or abnormal types of glycogen in tissues. Their frequency is very low and they are considered rare diseases. Except for X-linked type IX, the different types are inherited in an autosomal recessive pattern. In this study we reviewed the literature from 1977 to 2020 concerning GSDs, biomarkers, and metabolic imbalances in the symptoms of some GSDs. Most of the reported studies were performed with very few patients. Classification of emerging biomarkers between different types of diseases (hepatics GSDs, McArdle and PDs and other possible biomarkers) was done for better understanding. Calprotectin for hepatics GSDs and urinary glucose tetrasaccharide for Pompe disease have been approved for clinical use, and most of the markers mentioned in this review only need clinical validation, as a final step for their routine use. Most of the possible biomarkers are implied in hepatocellular adenomas, cardiomyopathies, in malfunction of skeletal muscle, in growth retardation, neutropenia, osteopenia and bowel inflammation. However, a few markers have lost interest due to a great variability of results, which is the case of biotinidase, actin alpha 2, smooth muscle, aorta and fibroblast growth factor receptor 4. This is the first review published on emerging biomarkers with a potential application to GSDs.

## 1. Background

**Figure 1:**
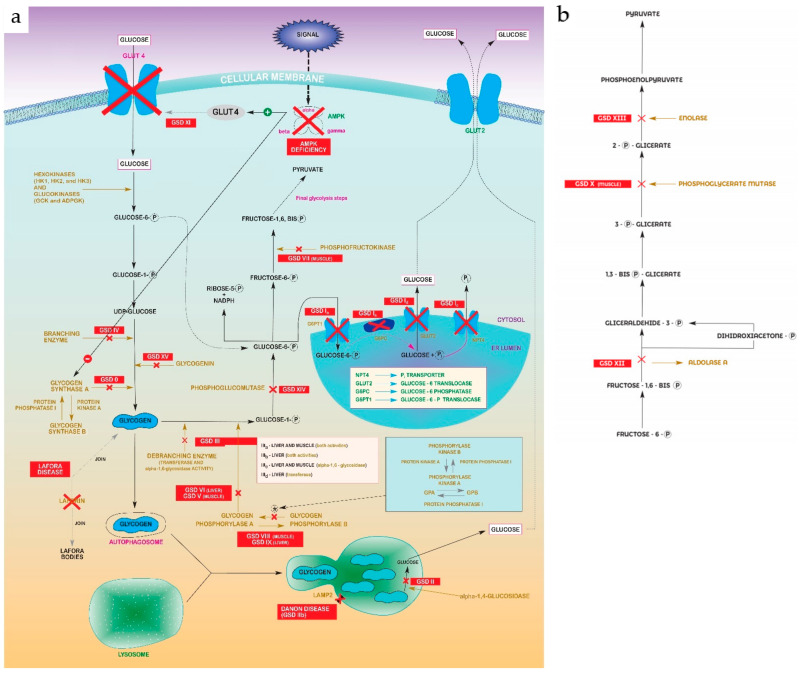
(**a**) Glycolysis–gluconeogenesis pathway showing steps disrupted in every GSD. Abbreviations: Glycogen Synthase Deficiency (GSD 0); Von Gierke Disease (GSD Ia, Ib, Ic, Id); Pompe disease (GSD II); Cori-Forbes Disease (GSD III); Andersen Disease (GSD IV); McArdle Disease (GSD V); Hers Disease (GSD VI); Phosphofructokinase Deficiency (GSD VII); Muscle Phosphorylase b Kinase Deficiency (GSD VIII); Liver Phosphorylase Kinase Deficiency (GSD IX); Fanconi-Bickel Syndrome (GSD XI); Phosphoglucomutase 1 Deficiency (GSD XIV); Glycogenin Deficiency (XV). (**b**) “Zoomed in” detail of pyruvate formation in final glycolysis steps from Figure 1a. Abbreviations: Muscle Phosphoglycerate Mutase Deficiency (GSDX); Aldolase A Deficiency (GSDXII); Enolase 3 Deficiency (GSDXIII).

Glycogen storage diseases (GSDs) are metabolic disorders of glycogen metabolism. In aggregate, GSDs are not considered rare diseases, but every individual type are. The overall estimated GSD incidence is 1 case per 20,000–43,000 live births [1]. There are 19 types, which are classified by enzyme deficiency and affected tissue. Most of them are inherited in an autosomal recessive manner, except for one X-linked GSDIX subtype. Disorders of glycogen degradation, shown in Figure 1a,b, may primarily affect the liver (hepatic GSDs), the muscles (muscle GSDs), or be multiorganic (mainly Pompe and Cori-Forbes disease) [2].

The most prevalent diseases are as follows:

1. Glycogen storage disease type I (GSDI, Von Gierke disease) is characterized by the accumulation of glycogen and fat in the kidneys and liver, resulting in renomegaly and hepatomegaly, which may give rise to a hepatocellular adenoma (HCA) or even to become malignant as hepatocellular carcinoma (HCC) [3]. The overall incidence of GSDI is 1:100,000. Affected neonates who are not medically treated have severe hypoglycaemia. Other children not treated present with hepatomegaly, lactic acidosis, hyperuricemia, hyperlipidaemia, hypertriglyceridemia, or hypoglycaemic seizures, usually from four months after birth. Another issue to consider is impaired platelet function, which can lead to bleeding and epistaxis. The variant GSDIb shows an impaired neutrophil and monocyte function and chronic neutropenia, in addition to complications mentioned before. This leads to recurrent bacterial infections and oral and intestinal mucosal ulcers. In general, untreated GSDI quickly leads to multiple complications. Most affected individuals live to adulthood. GSDI is diagnosed by identifying biallelic pathogenic variants:
  - GSDIa, caused by a deficiency of glucose-6-phosphatase (G6Pase or G6PC1) catalytic activity;
  - GSDIb, GSDIc and GSDId, caused by different defects in the glucose-6-phosphate transporter (SLC37A4).
2. Glycogen storage disease type II (GSDII, Pompe disease), with an incidence variability depending on ethnicity and geographic region, from 1:14,000 in African Americans to 1:100,000 in individuals of European descent, is classified by age of onset, organ involvement, severity, and rate of progression [4]:
  - Infantile-onset Pompe disease (IOPD); in children younger than 12 months, the clinical manifestations include cardiomyopathy, which may already be evident in the uterus. However, the most typical onset of the disease occurs at around 4 months. In this case, patients most frequently present with hypotonia, a muscle weakness that occurs in a generalized manner, and hypertrophic cardiomyopathy. These patients usually have serious difficulties feeding and breathing. If not treated with enzyme replacement therapy (ERT), patients with IOPD usually die after two years from progressive left ventricular outflow obstruction and respiratory insufficiency.
  - Late-onset Pompe disease (LOPD; including (a) individuals with onset before the age of 12 months without cardiomyopathy and (b) all individuals with onset after the age of 12 months) is characterized by proximal muscle weakness and respiratory insufficiency. Clinically significant cardiac involvement is uncommon.

The diagnosis of GSDII is established in a proband with either deficiency of acid alpha-glucosidase enzyme (GAA) activity or biallelic pathogenic GAA variants based on molecular genetic testing.

3. Glycogen storage disease type III (GSDIII, Cori-Forbes disease), with an incidence of 1:100,000 too, is characterized by variable liver, cardiac muscle, and skeletal muscle involvement. GSDIIIa is the most common subtype, present in about 85% of affected individuals [5]. It manifests with liver and muscle involvement. GSDIIIb, with liver involvement only, comprises about 15% of all GSDIII. The clinical significance of GSDIII ranges from almost no symptoms to severe cardiac dysfunction, congestive heart failure, and, rarely, sudden death. Skeletal myopathy manifests as muscle weakness but is rarely evident in childhood because it progresses slowly. This acquires greater relevance in the third to fourth decades of life. The diagnosis is made by identifying biallelic pathogenic variants in glycogen debranching enzyme, amylo-alpha-1,6-glucosidase, or 4-alpha-glucanotransferase (AGL).
4. Type V glycogen storage disease (GSDV, McArdle’s disease) is a metabolic myopathy whose main characteristic is exercise intolerance. Incidence roughly reaches 1:100,000 [6]. Symptomatologically, patients present with rapid onset fatigue, myalgia, and muscle cramps [7]. The symptomatology is more acute with exercise, and symptoms are usually isometric or sustained aerobic exercise. It is common for patients to experience greater exercise by exploiting the “second wind” phenomenon, which relieves myalgia and fatigue after resting for a few minutes. Normally GSDV symptoms appears in the first decade of life. Twenty-five percent of patients will present with weakness that mainly affects the proximal muscles. The diagnosis is made using PYGM molecular genetic tests (which encode glycogen phosphorylase, muscle form). This is the only known gene associated with GSDV.
5. Glycogen storage disease types VIII and IX (GSDVIII and GSDIX), results from a liver phosphorylase b kinase enzyme deficiency, which plays an important role in the decomposition of glycogen [8]. One type is hepatic PhK deficiency (GSDIX), characterized by the presence of early-onset hepatomegaly. There is also growth retardation and, often, fasting ketosis and hypoglycaemia. Another variant is muscle PhK deficiency (GSDVIII), much less common and related to physical exercise intolerance, myalgia, muscular cramps, myoglobinuria, and progressive muscular weakness. The PhK enzyme comprises four subunits (α, β, γ, and δ). Pathogenic variants of PHKA1, which encodes the α subunit, cause the rare muscular deficiency of the X-linked disorder. The pathogenic variants of PHKA2, which also codes for the α subunit, are responsible for the most frequent variant, hepatic PhK deficiency (X-linked hepatic glycogenosis). Incidence for GSD IXα is about 1:100,000 individuals [9]. Pathogenic variants of PHKB, which encodes the β subunit, cause PhK deficiency in both the liver and muscle. Those of PHKG2, which encodes the γ subunit, cause PhK deficiency in the liver. The diagnosis is enhanced by clinical findings from assessing the activity of PhK in erythrocytes or the liver or muscle tissues (depending on the variant to be treated), and definitive confirmation from molecular genetic testing.

Efforts to establish clinical biomarkers associated with GSDs were limited until recently. In Pompe disease (PD), serum biomarkers of tissue damage such as creatine kinase (CK), creatine kinase-muscle/brain (CK-MB), aspartate aminotransferase (AST), alanine aminotransferase (ALT), and lactate dehydrogenase (LDH) are usually used in laboratory tests, but they are nonspecific and have limited prognostic value. The direct measurement of muscle glycogen for diagnosis and monitoring requires invasive and sometimes risky biopsies and has limited sensitivity for late-onset forms of the disease because of the heterogeneity in the distribution of glycogen deposition [10,11]. For hepatic forms, parameters such as lactic acid, uric acid, 25(OH)-vitamin D, cholesterol, triglycerides, aspartate aminotransferase (AST), alanine aminotransferase (ALT), and gamma-glutamyltransferase (GGT) are used to monitor the normal control of the disease. For muscle forms, the main biomarker is creatine kinase (CK). In recent years, many studies have emerged looking for new biomarkers to improve the diagnosis and prognosis of different GSDs.

This work is an attempt to compile the first review focusing on biomarkers related with GSDs in aggregate. The literature search was not performed only once but involved more than 10 years of collaborations with rare diseases and GSD patient associations.

## 2. Materials and Methods

**Figure 2:**
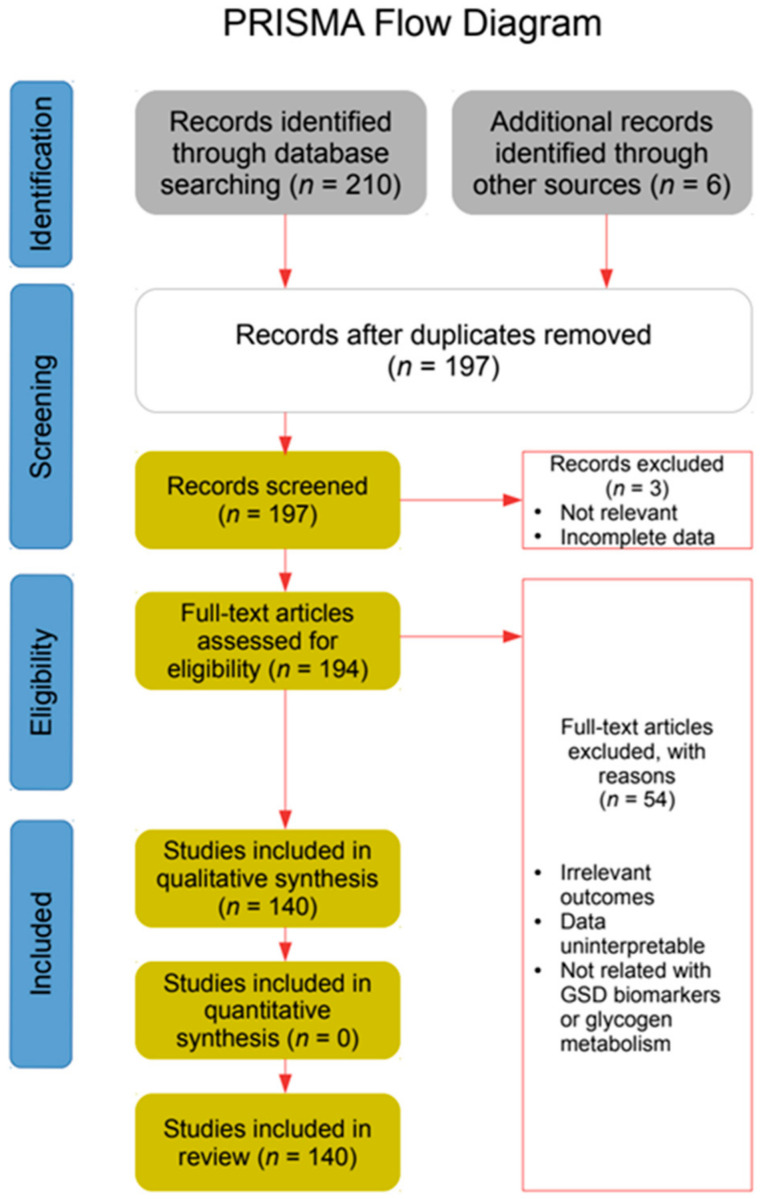
PRISMA flow diagram of bibliographic revision.

Public resources such as Pubmed, ScienceDirect, Google Scholar, and Orphanet were searched extensively for articles published from 1970 to January 2020. Keywords such as GSD (in short and extended form), glykogenose, glycogen, Von Gierke, Pompe, Cori-Forbes, Andersen, Danon, McArdle, Tarui, Polyglycosan, Hers, and gene names from each of the GSDs were used. Information obtained from International (Heidelberg 2013, Lyon 2012 and Milano 2010) and Spanish (from 2003 to 2015) GSD conferences was consulted as well (see Figure 2 for detailed diagram of searching process according to PRISMA statement [12]). In this review, biomarkers are classified according to those clinical and pre-clinical studies tested on human samples (“human studies”) and those showed on laboratory animals but not yet proved on human samples (“animal studies”).

## 3. Markers Found in Von Gierke Disease (GSDI)

### 3.1. Hepatic Steatosis, Hepatocellular Adenoma, and Carcinoma in GSDI

#### 3.1.1. Human Studies

As mentioned in the background, two of the most serious complications in GSDI are HCA and HCC. Regarding to HCA markers, Calderaro et al. [13] showed a molecular profile in GSDI-related HCA (H-HCA) characterized by a lack of hepatocytic nuclear factor 1 alpha (HNF1A) inactivation. A series of 25 tumor and 7 non-tumor frozen samples from 15 patients (age range 17–43 years old) with GSDIa (*n* = 14) and GSDIb (*n* = 1) were collected from 2004 to 2012. GSD-associated HCA was inflammatory (IHCA: 52%, 13/25), β-catenin activated (bHCA: 28%, 7/25) or unclassified (UHCA: 20%, 5/25). Among IHCA, interleukin 6 signal transducer (IL6ST), guanine nucleotide binding G protein alpha stimulating (GNAS) complex locus, and signal transducer and activator of transcription 3 (STAT3) mutations were found in 54% (7/13), 15% (2/13), and 0% (0/15) of the cases, respectively. In two tumors, β-catenin (CTNNB1) and IL6ST mutations were observed and classified as bHCA. In all cases, the mutations were heterozygous and somatic. All the mutations in IHCA were also predicted to activate glycoprotein 130 (gp130) or GNAS, leading to the activation of the IL-6/JAK/STAT3 pathway independently of the ligand [14]. All analyzed samples with either IL6ST or GNAS mutations showed high C-reactive protein (CRP) and/or serum amyloid A2 (SAA) expression. It is noteworthy that four mutations were classically located in CTNNB1 exon 3 in the bHCA cases. However, three were unusual and located in exon 7, previously described as very rare in HCC [15]. It is also remarkable that 6 out of the 7 bHCA cases with a mutation in the CTNNB1 gene showed high expression of a leucine-rich repeat containing G protein-coupled receptor 5 (LGR5) and/or glutamate-ammonia ligase (GLUL), two genes classically activated by β-catenin [16]. On the other hand, GSD non-tumor livers demonstrated metabolic defects characterized by [17]:

- Gluconeogenesis repression: decreased expression of phosphoenolpyruvate carboxykinase 1 and 2 (PCK1 and PCK2), glucose-6-phosphate transporter 1 (G6PT1), and fructose-1,6-bisphosphatase (FBP1);
- Glycolysis activation: increased expression of glucokinase (GCK) and glucose-6-phosphate isomerase (GPI), and decreased expression of glucokinase regulatory protein (GCKR);
- Fatty-acid synthesis activation: increased expression of acetyl-CoA carboxylase-α (ACACA), ATP citrate lyase (ACLY), fatty acid synthase (FASN), malic enzyme 1 (ME1), and fatty acid desaturase 1 and 2 (FADS1 and FADS2).

This expression profile was very similar to that observed in HNF1A-inactivated HCA but markedly different from that in steatotic and non-steatotic non-tumor liver tissues. Moreover, hierarchical unsupervised clustering showed that the non-tumor GSD livers clustered in the same group as the H-HCA samples. As a summary, Calderaro and colleagues identified β-catenin-activated HCA as potentially related to malignant transformation and found common metabolic defects in HCA inactivated for HNF1A and in GSD non-tumor livers.

A cohort of 55 GSDIa patients aged 2–39 years (25 women and 30 men) was established in another study [18]. The control group consisted of 28 individuals in the same age range. Fourteen patients in the study group (eight men and six women) had HCA. The peripheral blood of GSDIa patients with (Group 1) and without HCA (Group 2) was compared to that of the control subjects; the levels of the CXC chemokine interleukin-8 (IL8) were 1.4 times higher in the Group 2 patients than in the controls, and 2.8 times higher in Group 1 than in the controls. There was a correlation between the levels of circulating IL8 and the severity of liver damage [19,20]. IL8 levels increased in those with GSDIa, and the high neutrophil count in the peripheral blood corresponded to those seen in mice affected by GSDIa and analyzed in same study [18]. The authors supposed that glycogen accumulation in the liver produces inflammation and that the accumulation of lipids in this organ can stimulate the production of IL8, a sign of chronic liver inflammation that can lead to liver damage. ChREBP/mammalian target of rapamycin (mTOR) activation and AMP-activated protein kinase (AMPK) inhibition [21,22] also represent likely key molecular events orchestrating metabolic reprogramming in GSDI during liver adenoma or carcinoma progression [23].

Another study [24] compared genetic alterations in a group of 17 patients (young and adult individuals) with HCA, 10 of whom had GSDI and 7 did not, by PCR amplification and sequencing of peripheral blood, liver adenomas and the corresponding non-neoplastic tissues. Chromosomal aberrations were found in 60% of the patients with GSDI and 57% of the others. Notably, authors proposed that in those HCAs associated with an increase in insulin-like growth factor 2 (IGF2) by inactivation of the insulin-like growth factor 2 receptor (IGF2R) gene, a therapy targeting IGF2 could effectively prevent tumor malignancy. With this evidence, IGF2 could be proposed to distinguish between HCA and hepatocellular carcinomas (HCC) in GSD patients.

Using massively parallel sequencing, miRNA profiling was performed on paired adenomas and normal liver tissues from seven young and adults GSDIa patients [25]. Differentially expressed miRNAs were validated in liver tumor tissues, CHC cell lines, and serum by quantitative RT-PCR. It was shown that miR-130b can be used as a circulating biomarker for the detection of GSDIa HCA. Likewise, miR-130b, miR-32, miR-199a-5p, miR-199b-5p, miR-214, and miR-582-5p could be useful for monitoring the malignant transformation of HCA. miR-224 and miR-452 could be used as therapeutic biomarkers, since they are highly overexpressed in GSDIa HCA, general-population HCA, and HCC cell lines.

Another proposed biomarker in HCA is hepcidin (HEPC). Weinstein and colleagues [26] proved a role for this marker in a remarkable iron refractory anemia observed in a cohort of GSDIa patients (1 child and 4 adults). Side-by-side comparison in liver samples from 2 adult patients showed that HEPC mRNA expression in unaffected liver tissue from both patients was significantly lower than in the adjacent HCA tissue. Authors suggested a central role of HEPC in iron homeostasis regulation and acts as a critical mediator in the anemia of GSDIa.

#### 3.1.2. Animal Studies

Kim et al. [27,28] showed that non-tumor-bearing (NT), recombinant adeno-associated virus (rAAV) vector-treated GSDIa mice (AAV-NT mice) expressing various levels (0.9–63%) of normal hepatic glucose-6-phosphatase-α had active hepatic AMP-activated protein kinase (AMPK)/sirtuin-1 (SIRT1) signaling pathways after 90 weeks of treatment, unlike untreated mice. AMPK suppresses inflammation by inhibiting the interleukin-6 (IL-6)-mediated phosphorylation and activation of STAT3 signaling [29]. The levels of IL-6 and phosphorylated forms of the STAT3 protein at the Y705 position (p-STAT3-Y705) in the AAV-NT mice were lower than those in control mice. Moreover, SIRT1 inactivates NF-κB, a transcription factor that regulates inflammation and promotes inflammation-associated cancer [30], by deacetylating the K310 residue of the NF-κB p65 subunit, as was found in human cell lines from non-small-cell lung cancer, immortalized lung epithelium [31], and normal gastric epithelium [32]. Thus, the STAT3 and NF-κB signaling pathways are downregulated in AAV-NT mice, protecting them from HCA/HCC, as the authors suggested. Moreover, SIRT1 is a negative regulator of tumor metastasis that upregulate E-cadherin and downregulate the mesenchymal markers, N-cadherin and vimentin levels [33]. AMPK and SIRT1 are two modulators involved in the regulation of energy metabolism. This fact assumes that genes such as fibroblast growth factor 21 (FGF21) [34] and its tumor suppressor co-receptor β-klotho [35] are activated. All these molecules (AMPK/SIRT1/FGF21/β-klotho) and the downregulation of the STAT3/NFκB signaling pathways provide the AAV-NT mice with a physiology that hinders tumor formation. When AAV-NT mice were treated with 6-chloro-2,3,4,9-tetrahydro-1H-carbazole-1-carboxamide (EX-527), a selective and potent inhibitor of SIRT1 [36], the anti-inflammatory and anti-tumor properties of SIRT1 were reversed. The hepatic levels of β-klotho were similar in the AAV-NT mice before and after treatment with EX527, so the activation of β-klotho is independent of SIRT1 activity.

Continuing with the analysis of SIRT1, Cho et al. [37] used adult L-G6PC−/− mice to discover an important role of SIRT1 in maintaining autophagy in G6Pase-α-deficient livers (origin of GSD1a in humans). They showed that hepatic G6Pase-α deficiency altered the activity and/or expression of several lipid regulators, leading to hepatic steatosis and reduced expression of SIRT1. The downregulation of SIRT1 increased the acetylation of ATG proteins—critical for autophagic vesicle elongation—and reduced the activity of FOXO factors that can induce autophagy genes. Remarkably, hepatic NAD+ levels were unchanged. Suppressed expression of peroxisome proliferator-activated receptor-α (PPAR-α)—a master regulator of fatty acid β-oxidation—and increased hepatic G6P, which activates signaling via the transcriptional activator and repressor ChREBP, were also observed [38]. Thus, G6Pase-α-deficient mouse livers displayed impaired autophagy, impaired autophagosome formation, and reduced autophagic flux. Based on these results, Cho and colleagues proposed that the transcription of SIRT1 could be stimulated by PPAR-α and downregulated by peroxisome proliferator-activated receptor-γ (PPAR-γ) and carbohydrate responsive element binding protein (ChREBP) [39]. Notably, the G6P-mediated activation of hepatic ChREBP signaling was reversed using rAAV-G6PC-mediated gene therapy to restore hepatic G6Pase-α expression. So, an important role of SIRT1 in maintaining autophagy function in G6Pase-α-deficient liver has been shown, giving rise to a new good candidate for prognostic biomarker.

### 3.2. Observed Predictor of Metabolic Control in GSDI

#### Human Studies

IGF2 was also identified as a predictor of metabolic control in young patients with GSDI. Melis et al. [40] in a prospective case-control study, enrolling 10 GSDIa and 7 GSDIb patients with dietary treatment, compared to 34 control subjects, demonstrated a significant difference in IGF2 levels between GSDI and control subjects, in peripheral blood mononuclear cells. Lack of alterations in the IGF2 gene region rules out genetic factors as an explanation for the increased IGF2 levels. Overall, authors hypothesized that the improved metabolic control in GSDIa patients caused by a specific dietary treatment increased insulin secretion, responsible for the reduction of GH secretion and the increase in circulating IGF2 levels.

### 3.3. Inflammatory Bowel Disease in GSDI

#### Human Studies

Populations with GSDI suffer from Crohn’s-like inflammatory bowel disease (C-IBD) [41]. The evaluation and risk stratification of patients according to calprotectin levels in the faeces represent a non-invasive and cheap test [42]. Calprotectin is a calcium-binding protein mainly found in neutrophils and belongs to a subgroup of proteins in the S100 family. The expression of calprotectin is suggested to be limited to early stages of myeloid differentiation, since it is present in circulating granulocytes, monocytes, and macrophages. Phagocytes expressing calprotectin belong to the first cells infiltrating inflammatory lesions. Neutrophils have been found in patients with inflammatory bowel disease (IBD), supporting the hypothesis that the increase in fecal calprotectin is initially a result of increased neutrophil migration into the gut lumen across inflamed mucosa. Apart from anti-infective host-defense roles, it has important proinflammatory properties, and high concentrations can be found during inflammation in the serum and at sites of infection [43,44,45,46,47]. Median faecal calprotectin levels were significantly higher in patients with IBD than in other patients (89% sensitivity and 79% specificity). This difference was greater than for C-reactive protein (CRP), the erythrocyte sedimentation rate (ESR), or a combination of both. Higher faecal calprotectin levels were reported in IBD than non-IBD patients, translating into an excellent mean sensitivity and specificity of 95% and 91%, respectively. Using faecal calprotectin for screening, there was a 67% reduction in endoscopy requirements for enrolled patients. This capacity of screening was superior to CRP, ESR or serological markers such as anti-neutrophil cytoplasmatic antibody (ANCA) and anti-Saccharomyces cerevisiae antibody (ASCA).

Another advantage of calprotectin to reflect inflammatory conditions is given because it is far more precise when measured in faeces than other biomarkers measured in the serum. In clinical practice, faecal calprotectin is a marker of mucosal damage that apparently fluctuates depending on disease location. The recommended cut-off level for an abnormal test result is 50 μg of calprotectin per gram of faeces but could be changed depending on the clinical situation [42]. Unfortunately, faecal calprotectin seems to not be reliable as a marker of IBD in glucose-6-phosphatase catalytic subunit 3-deficient (G6PC3) patients, a congenital disease which shares chronic neutropenia with GSDIb. Normal levels of this marker were noted during times of neutropenia and IBD, resulting in false negative tests [48]. Its value for diagnosis in different clinical settings warrants further study.

### 3.4. Neutrophil Impairment in GSDIb

#### 3.4.1. Human Studies

Regarding neutropenia in GSDIb, which involves mutations in the glucose-6-phosphate translocase (G6PT), Veiga da Cunha et al. [49] proved recently that it is caused by excessive accumulation of a nonclassical metabolite with unknown physiological role, 1,5-anhydroglucitol-6-phosphate (1,5AG6P), which results from the phosphorylation of 1,5-anhydroglucitol (1,5AG), also called 1-deoxyglucose, a glucose analog normally present in blood. This phenomenon was also seen in G6PC3 deficiency, which causes symptoms such as neutropenia, neutrophil dysfunction with malformations in patients affected, but has no impact on glucose homeostasis. This metabolite inhibits the first step of glycolysis by inhibiting low-K_M_ hexokinases. This is particularly detrimental in neutrophils since their energy metabolism depends almost entirely on glycolysis. Treatment with a sodium-glucose transport protein subtype 2 (SGLT2) inhibitor released the blockage and allowed neutrophil maturation [50]. Five GSDIb patients (3 children and 2 adults) were treated with a SGLT2 inhibitor and improved their heterogeneous clinical findings related to neutrophil dysfunction [50,51]. Based on this, 1,5AG could be a good candidate to be a biomarker for treating neutropenia in GSDIb and G6PC3-deficient patients.

#### 3.4.2. Animal Studies

In addition, Jun et al. [52], using GSD-Ib (G6pt−/−) mice, showed that the hypoxia inducible factor 1 subunit alpha (HIF-1α)/peroxisome proliferator-activated receptor-gamma (PPAR-γ) pathway directly impacts neutrophil chemotaxis, respiratory burst, and calcium mobilization in deficient neutrophils.

On the other hand, Kim and colleagues [53] showed that the levels of the β2 integrin α subunits, CD11b and CD11a, in neutrophils from G6PT−/− mice, were significantly decreased compared to those in neutrophils from control mice. This suggested that G6PT deficiency may cause the deglycosylation of CD11b and demonstrated that the underlying cause of impaired neutrophil migration and adhesion in G6PT-deficient mice arises from the aberrant expression of CD11b and CD11a.

### 3.5. Osteopenia and Osteoporosis in GSDI

#### 3.5.1. Human Studies

It is well known that suboptimal metabolic control, associated with a variable grade of ketosis, may play a role in reduced bone mass density (BMD). A high protein intake may contribute to ketosis as well. One additional factor is the endocrine imbalance, involved in osteopenia. In this case, IGF1 regulates bone growth and enhances osteoblast proliferation [54]. Insulin-like growth factor type 1 (IGF1) was also proposed as an early marker of osteopenia/osteoporosis [55]. Approximately 99% of circulating IGF1 is normally bound to IGFBPs 1–6 [56]. Therefore, IGFBPs modulate the availability of IGF1 for binding receptors. Among these, insulin-like growth factor binding protein 3 (IGFBP3) is the most abundant protein, providing 80% of the IGF1-binding capacity in the serum [57] and its increase reduces the amount of IGF1 available to bind receptors. It was suggested that the molar ratio of IGF1 to IGFBP3 could be used as an indicator of IGF1 bioavailability [58]. A correlation between BMD and IGF1/IGFBP3 ratio in peripubertal females has also been shown [59]. On the other side, nutritional status can directly affect IGF1 serum levels. Particularly low protein intake is associated with decreased IGF1 serum levels [60,61].

#### 3.5.2. Animal Studies

It was noted that IGF1 mediates PTH’s effects on bone in mice [62] and it has been shown that it promotes osteoblastic activity by activating the mTOR pathway [63].

### 3.6. Potential Diagnostic Marker in GSDI

#### Human Studies

Kurbatova et al. [64] analyzed lymphocyte mitochondrial enzyme activity. They revealed a significant decrease in succinate dehydrogenase (SDH), most expressed in GSDI. They also noted a tendency towards increased NADH-dehydrogenase (NADH-H), indicating compensatory respiratory chain I phase activation and II phase depression, and a significant decrease in glycerol-3-phosphate-dehydrogenase (GPDH), indicating a loss of glycolysis and Krebs-cycle coupling, when comparing 86 children with hepatic GSD (35 type-I, 23 type-III, and 27 type-VI) to a control group of 34 healthy children. The authors proposed the use of these laboratory parameters as additional diagnostic data to assess the state of children diagnosed with GSD.

### 3.7. Unreliable Markers

#### Human Studies

Three studies disregarded certain biomarkers due to different inconsistencies:

- The enzyme biotinidase (BTD) catalyzes the hydrolysis of biocytin to biotin and is mostly produced in the liver. Its serum levels in patients with GSDI were evaluated according to Paesold-Burda et al. [65]. The normal levels of biotinidase in healthy people fall between 7.0 and 10.6 mU/mL.
- All GSDI patients analyzed showed elevations of these levels. In the case of GSD type I non-a, the values were increased 2.4 times. The sensitivity was 100% for GSDI patients. No specificity data was found in this study. In addition, no correlation was observed between the activity of BTD and age or dietary control. The enzyme’s activity was also independent of the liver glycogen content. However, another study by Angaroni et al. [66] revealed that not all GSDIa are linked to increased BTD activity, in contrast to previous studies [65,67,68,69]. The authors observed oscillating enzymatic values in GSDIa patients. In addition, they suggested that the presence of a functional polymorphism could influence the expression of this biomarker. The intra- and inter-individual variability of the BTD activity in the GSD individuals suggested that it had little value as a potential biomarker.
- One study on the importance of bone mineral density turnover in patients with hepatic GSDs concluded that some patients with GSDI had increased bone resorption, implied by increased urinary free pyridinoline (fPYD)/creatinine and free deoxypyridinoline (fDPD)/creatinine ratios and serum concentration of the carboxy-terminal telopeptide of type I collagen (ICTP). However, the authors found no correlation between any markers of bone destruction and total body z-score [70].
- After observing a sample of 8 patients (6 males, 2 females) with hepatocellular carcinoma (HCC) and GSDIa, one group suggested that α-fetoprotein (AFP) and carcinoembryonic antigen (CEA) do not appear to be reliable indicators of the presence of hepatic malignancy in patients with GSDI [71].

**Figure 3:**
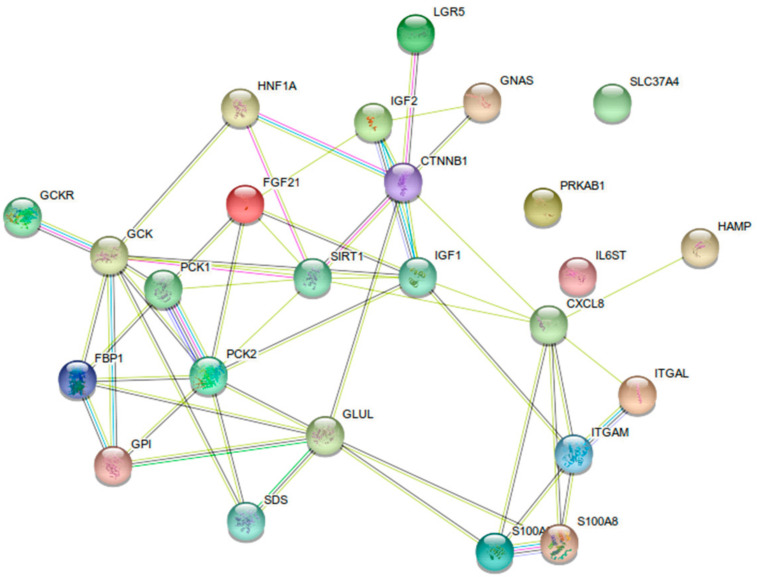
Protein-protein interaction map of possible biomarkers for GSDI extracted from Table 1 using STRING.

The STRING database [72] was used to visualize interactions between the biomarkers mentioned in Table 1 (see Figure 3). The central role of proteins such as SIRT1, IGF1, GSK3B (glycogen synthase kinase 3β), HIF1A (hypoxia inducible factor 1 subunit alpha), GCK, and PCK2 in the interaction network influencing hepatic GSDs is remarkable.

## 4. Putative Markers Found in Other Hepatic GSDs

### 4.1. Urinary Marker

#### Human Studies

In the search for more markers to improve the early differential diagnosis between GSDI, GSDIII, and GSDIX, whose clinical characteristics were already mentioned in the background, urinary glucose tetrasaccharide, also known as Glc4 (Glcα1-6Glcα1-4Glcα1-4Glc), emerge as a new possibility. Glc4 is a limit dextrin produced by the amylolytic digestion of glycogen and other branched glucose polymers such as amylopectin that contain α-1-6 glycosidic linkages [74,75]. It has been found in erythrocytes of all the GSDIII patients (57 patients, 137 blood samples), as commented recently by Piraud et al. [76]. Specially increased for patients older than 20 years (*p* ≤ 0.001). These results are comparable with one GSDIII patient (out of 6) reported by Sidbury and colleagues [77]. In addition, Glc4 was markedly increased in GSDIII patients, aged ≤22 years and adults [78], and in some patients with GSDIX aged <18 years [79].

As an important recommendation, Glc4 was proposed as a new biomarker for liver fibrosis and cirrhosis in a pediatric GSDIII cohort [80]. Glc4 is derived primarily from hepatic glycogen, and its decrease may be secondary to fibrosis progression leading to cirrhosis. A correlation was seen for Glc4 with AST and ALT, but not CPK. Moreover, in another study, which managed an hepatic GSD cohort of 79 individuals, it was suggested that in pediatric patients, decreasing Glc4 excretions reflect improved fasting tolerance (i.e., a liver function), whereas in adults, Glc4 excretion may be associated with chronic, progressive skeletal muscle involvement [81]. This trends in urinary Glc4 and its correlation with the transaminases were comparable with other trends observed in four GSDIIIa dogs, similar to findings for human GSDIIIa [82].

### 4.2. Cardiomyopathy in GSDIII

#### Human Studies

Troponin (Tn) contains three subunits, Ca^2+^ binding (TnC), inhibitory (TnI), and tropomyosin binding (TnT). TnI is a structural protein with three isoforms: one cardiac isoform (TNNI3) and two skeletal isoforms (sTnI), called slow skeletal TnI (TNNI1) and fast skeletal TnI (TNNI2). TnI is a well-known marker of myocardial injury [83] that may have utility as a plasma marker for skeletal muscle damage [84]. The skeletal isoforms have approximately 40% sequence dissimilarity between the skeletal and heart isoforms [85], and there is no sTnI in the adult heart [86,87]. An increase in plasma sTnI also indicates severe damage. B-Type natriuretic peptide (BNP) is a cardiomyocytes-secreted hormone located in the heart ventricles in response to cardiac stress and ventricular dysfunction [88]. Several studies have demonstrated the BNP prognostic value in patients with myocardial involvement.

In a study which examined cardiac tissue in three adult GSDIII patients [89], authors found cardiac fibrosis (1 patient), moderate to severe vacuolation of cardiac myocytes (3 patients), mild to severe glycogen accumulation in the atrioventricular node (3 patients), and glycogen accumulation in smooth muscle cells of intramyocardial arteries associated with smooth muscle hyperplasia and profoundly thickened vascular walls (1 patient). Troponin level (no subunit neither isoform specified by the authors) was significantly elevated in patients who suffered a progressive hypertrophic cardiomyopathy with systolic heart failure, that became more symptomatic with severe dyspnea on exertion, worsening fatigue, 3-pillow orthopnea, and chest tightness with slight exertion. In the rest of the patients, troponin levels were not obtained. No more evidence for the use of Troponin nor BNP as cardiac involvement markers was found in the literature; except for in a work that showed the application of an intravenous form of BNP (nesitiride) as treatment for patients with acute decompensated heart failure [90]. More research is necessary to fill the gap of troponin and/or BNP as useful markers of cardiac involvement in GSDIII.

### 4.3. Osteopenia and Osteoporosis in GSDIII

#### Human Studies

Another proposed factor in BMD reduction is hyperlipidemia, which could be influencing the decreased BMD in GSDIII patients. Regarding IGF1 as a diagnostic factor, Melis et al. [73] confirmed a correlation between the BMD and IGF1/IGFBP3 ratio and suggested that reduced IGF1 levels may contribute to reduced BMD in GSDIII patients (aged 2–20 years). In addition, the lower IGF1 serum levels detected could be poorly related to the GSDIII patient nutritional status, owing to frequent feedings provided to this kind of patients, as well as a high protein intake [73].

### 4.4. Unreliable Markers

#### Human Studies

Biotinidase (BTD) serum levels were also evaluated in other types of GSD (III, VI, IX, and XI) [65]. All other types of GSDs analyzed showed elevations of these levels too. In the case of GSD type III and XI, the values were increased 1.5 and 1.8 times, respectively. The sensitivity was 100% for GSDVI and XI patients, 62% for GSDIII, and 77% for GSD IX. The authors observed oscillating enzymatic values in GSDIII, and GSDIX patients as well. Due to these observations, BTD is not yet a reliable diagnostic biomarker for hepatic GSDs.

**Figure 4:**
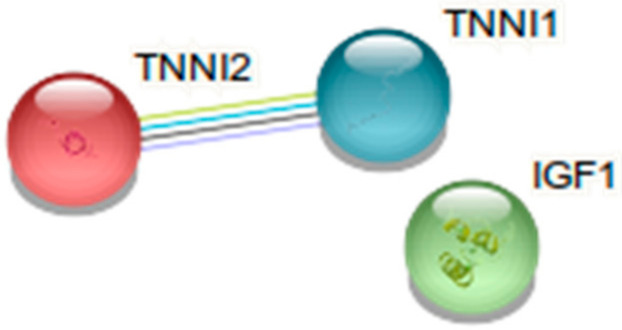
Protein-protein interaction map of possible biomarkers for other hepatic GSDs extracted from Table 2 using STRING.

The STRING database [72] was used to visualize interactions between the biomarkers mentioned in Table 2 (see Figure 4). Only troponins (TNNI1 and TNNNI2) has a relationship between them.

## 5. Markers in Pompe Disease (GSDII)

### 5.1. Malfunction of Skeletal Muscle in GSDII

#### 5.1.1. Human Studies

Palermo et al. [91] performed a preliminary search for factors that define Pompe disease (PD), whose clinical aspects are summarized in the background, and those that distinguish it from other lysosomal storage diseases (LSDs) and myopathies in 11 pediatric patients. Differences in gene expression in quadricep and bicep tissues between infantile-onset PD individuals (IOPD) and healthy people were observed. Myosin heavy chain 3 (MYH3), myosin heavy chain 8 (MYH8), myogenin (MYOG), myogenic factor 5 (MYF5), myocyte enhancer factor 2B (MEF2B), myocyte enhancer factor 2C (MEF2C), cardiac troponin T2 (TNNT2), acetylcholine receptor alpha subunit (CHRNA1), and ankyrin repeat domain 1 (ANKRD1) were among the genes whose expression was remarkably increased in IOPD. The expression of actin alpha 2, smooth muscle, aorta (ACTA2) was upregulated, and fibroblast growth factor receptor 4 (FGFR4) was unchanged in the biceps. Meanwhile, decreased expression was observed in the quadriceps for ACTA2 and FGFR4. Some transcription factors of immature muscle cells or undifferentiated muscle cells such as transforming growth factor beta 1 (TGFβ) and tumor necrosis factor alpha (TNFα) were also upregulated in patients compared to controls. In addition, many gene sets associated with inflammation and the inflammatory response were differentially expressed in the IOPD patients. The gene that codes for the B-cell antigen receptor complex-associated protein alpha chain (CD79A), a marker of B cells, is elevated in IOPD and is consistently differentially expressed in the comparison of positive with poor clinical outcomes.

Studying the overlapping genes, it was observed that the genes upregulated in the patient samples were similar to those expressed in:

- Immature or regenerating muscle cells (TNNT2; MYH3; myosin VA, MYO5A; and RUNX related transcription factor 1, RUNX1),
- Inflammatory processes (chitinase 3 like 1, CHI3L1; macrophage expressed 1, MPEG1; TIMP metallopeptidase inhibitor 1, TIMP1; and lipopolysaccharide binding protein, LBP) and
- Apoptosis (cyclin-dependent kinase inhibitor 1A, CDKN1A; PERP TP53 apoptosis effector; and prune homolog 2, PRUNE2).

A gene related to cardiomyopathy (ANKRD1) and another related to Duchenne muscular dystrophy (AHNAK nucleoprotein 2, AHNAK2) also showed increased expression in IOPD. It is convenient to highlight a group of 918 selected genes with altered expression in IOPD patients that could be used to evaluate the progression of the disease and the response to enzyme-replacement therapy (ERT). After 52 weeks of treatment with ERT, it was observed that the expression of the muscle genes in the patients with positive clinical results returned to levels closer to those found in the controls, which did not happen in the patients with poor clinical results. The ability to predict the progression of the disease increased if 918 genes were jointly analyzed, compared to the weak individual abilities. Authors hypothesized that a subset of these genes could serve as a good biomarker for disease severity in IOPD.

The differential protein expression of matrix metallopeptidase 2 and 9 (MMP2 and MMP9) was found in serum samples of two pediatric patients with PD, where an alteration in the expression of studied molecules was noted after the start of ERT. In addition, a differential expression was also found between PD patients and other LSDs. The authors [92] suggested that these molecules may be used as potential biomarkers for the diagnosis, follow-up and assessment of response to therapy in this group of disorders.

Another possible biomarker is lactosylceramide (LacCer), a molecule belonging to the glycosphingolipid group. Several studies describe the relationship between LacCer and the endosomal/lysosomal system, and this molecule increases in several tissues in some lysosomal storage diseases. In a study by Hůlková et al. [93], tissue samples from various body locations were used from both people affected by one of the lysosomal storage diseases and from healthy people (liver, heart, and skeletal muscle were used for PD pediatric patients). In the samples from people affected by PD, increases in LacCer storage were observed in both skeletal muscle cells and in cardiomyocytes compared to that in the control samples.

An interesting contribution was made by Pascual et al. [94], who measured the plasma levels of IGF1, IGFBP3, and growth hormone (GH) in 26 patients aged 15–72 years, including 15 females and 18 males. The levels of IGF1 and IGFBP3 gradually decrease with age. However, the results in these patients included significant elevations of both IGF1 and IGFBP3 compared to the corresponding reference ranges for the ages of three healthy groups (30–40, 41–50, and 51–70 years). GH levels were not relevant for all the patients.

#### 5.1.2. Animal Studies

Baligand and colleagues [95] observed that the phosphomonoester (PME) 31^P^ magnetic resonance spectroscopy (MRS) signal was elevated in the skeletal muscle of GAA−/− mice and throughout the lifespan of the animal (2, 6, 12, and 18 months of age). In addition, upregulating GAA activity via an AAV-based approach decreased the 31^P^ MRS PME signal. The authors asserted that these changes happened at the same time glycogen clearance was observed with increased GAA activity. These observations were showed by 13^C^ MRS, by 1^H^ high-resolution magic angle spinning of muscle samples, and by the biochemical determination of glycogen content in muscle extracts. Moreover, authors suggested that the changes in the PME signal detected in vivo could potentially be explained by changes in glucose-6-phosphate. Because 31^P^ MRS is inherently more sensitive than 13^C^ MRS, PME levels have high potential as a non-invasive clinical biomarker for metabolic changes associated with the progression and treatment of PD.

Defects in autophagy are one of the major characteristics of lysosomal-storage diseases such as PD. The overexpression of transcription factor EB (TFEB) leads to the production of new lysosomes and increases the number of autophagosomes in many cell types [96,97,98]. Using a PD knockout mouse model expressing lysosomal acid alpha-glucosidase (GAA−/−) with adenoviruses and plasmids as vectors, Spampanato et al. [99] found the following:

- Lysosomal exocytosis was observed in the myotubes in which TFEB was administered. This causes a significant decrease in the amount of accumulated storage material (in particular, glycogen).
- Confocal microscopy techniques showed that overexpression of TFEB did not damage muscle tissue and that muscle fibers treated with TFEB showed increased fusion of lysosomes and autophagosomes. If the autophagic process in the cells was suppressed, the effect of TFEB on lysosomal clearance was blocked. This indicates that the autophagic process is necessary for the TFEB-mediated lysosomal clearance.
- The levels of the transcription factor TFEB were about 10 times higher in mice treated with TFEB, and accumulated glycogen was almost completely cleared. In addition, the muscle ultrastructure underwent improvement due to a reduction in the size and number of lysosomes containing glycogen.

With these features (mainly, the promotion of cellular exocytosis and stimulation of lysosomal clearance in muscle-fiber cells), TFEB may be regarded not only as a promising prognostic biomarker but also as a breakthrough in the treatment of patients suffering PD.

The overexpression of a potential therapeutic biomarker, IGF1, accelerates the regeneration of skeletal muscle in mice [100] and in individuals after strength training. Myostatin (MSTN) was suggested to prevent the overgrowth of skeletal muscle, with muscle mass being higher in mice deficient in MSTN compared to normal mice [101]. Ten PD patients were recruited by Chien et al. [102], with both infantile-onset and late-onset disease, and the results were compared to those in 10 control patients in the same age range. Measurements of serum MSTN, IGF1, and CK levels were obtained. The levels of IGF1 and MSTN were measured in the PD patients before and after ERT. The levels of IGF1 and MSTN were lower in the PD patients before ERT than in control patients. After an average treatment period of 12 months, the MSTN levels in the PD patients increased by 129% and IGF1 increased by 74%. In the control patients, MSTN levels were practically the same after 12 months, while IGF1 levels were reduced. These results showed that IGF1 and MSTN may be related to muscle regeneration and correspond to the muscle recovery observed in these patients.

### 5.2. Urinary Biomarker in GSDII

#### Human Studies

Glc4 is a specific marker evaluated for IOPD in different studies. The urinary secretion of Glc4 seems to be the most important biomarker in measuring the progress of the disease. Glc4 is formed by intravascular degranulation of the glycogen released in circulation and corresponds to the degree of glycogen accumulation in the skeletal muscles of patients with PD. Two clinical trials, phase I/II [103] and phase II/III [104], were performed:

- The first trial showed that urinary and plasma Glc4 levels correlated with the motor response to treatment over 52 weeks for 11 infantile patients. Glc4 was measured by ultra-performance liquid chromatography-UV (UPLC-UV), to determine its levels in the urine of normal individuals (control group) and that of those with IOPD. This study found that changes in urinary Glc4 were related to the clinical response to ERT treatment. The urinary Glc4 levels were increased prior to treatment and decreased during treatment, normalizing or falling below the control range limits. After 2 months of treatment, they decreased and remained low during the first year of treatment. Therefore, the urinary levels of Glc4 correlated well with the clinical response to ERT.
- The second clinical trial was carried out with a different cohort of IOPD patients (*n* = 18) and described the correlation of urinary Glc4 with muscle glycogen content and motor function response to ERT over 2 to 3 years. The patients were classified into three groups. Group 1 (7 of 18 patients) had both the best motor function response in the initial phase and sustained clinical improvements by the end of the extension phase of the trial. Group 2 (6 of 18) showed measurable motor gains after 52 weeks of ERT but no further gains or suffered a clinical decline in the extension phase. Group 3 (5 of 18) failed to gain motor milestones at 52 weeks or by the end of the extension phase of the study and became invasive-ventilator dependent between 12 and 60 weeks of ERT. The results from this phase II/III clinical trial showed a strong correlation between urinary Glc4 and skeletal muscle glycogen at three different time points (0, 12, and 52 weeks). This latter trial also indicated that urinary Glc4 may have predictive value as a biomarker for IOPD patients. The value of Glc4 as a biomarker for monitoring the efficacy of treatment with serum enzyme markers of muscle damage is remarkable.

For these reasons, more studies were performed to determine the most efficient way of measuring Glc4 and yield more evidence of its utility as a biomarker:

- In the study conducted by Sluiter et al. [105], the direct separation of underivatized Glc4 from its tetrasaccharide M4 isomer via rapid ultraperformance liquid chromatography–tandem mass spectrometry assay (UPLC-MS/MS) exhibited a very high diagnostic specificity of about 92%. This suggested the urinary measurement of Glc4 to be a suitable biomarker for the early diagnosis of PD and for the clinical course of the disease after the administration of ERT. This study was performed with urine obtained from adults, children, and infants from 65 GSDII patients (not only IOPD), eight with GSDIII, two with GSDIV, and six with GSDIX. The authors proposed UPLC-MS/MS as a convenient technique for detecting and quantifying Glc4 as a urine biomarker in GSDII patients. The results also support the suitability of Glc4 as a marker for GSDIII [106,107] in pediatric patients. This method is fast and analytical, has good diagnostic sensitivity, and is safe within acceptable limits.
- Huang and colleagues [108] concluded that urinary Glc4 is very helpful for distinguishing between newborns with pseudo-deficiency of lysosomal acid alpha-glucosidase and those with IOPD. They observed that the level of urinary Glc4 decreased rapidly if the newborn received ERT within 4 h of admission. Unexpectedly low results were previously observed, despite stability studies indicating that urinary Glc4 should be stable for at least 7 days at room temperature (most likely due to degradation by bacterial action or excessive urinary enzyme accumulation secondary to ERT). However, salivary Glc4 can be analyzed, and researchers are looking to assess its efficacy as a potential biomarker for Pompe patients [109].
- Bobillo Lobato [110] analyzed concentrations of Glc4 using HPLC (high performance liquid chromatography) in urine samples from 35 patients affected by PD (9 with infantile presentation and 26 with adult presentation) by comparing them to 40 control patients. The concentrations of Glc4 were normalized to those of urinary creatinine. It was observed that the Glc4 values decreased with age in both the affected and control groups. A ROC curve was developed, exhibiting an area of 0.980 for the infantile form of PD. A value of 4.925 mmol/mol of creatinine was set as the cut-off point, with a sensitivity and specificity of 85.7%. For the adult form of the disease, the area under the curve was 0.949, and 1.025 mmol/mol of creatinine was set as the cut-off point, with a sensitivity of 89.3% and a specificity of 88.5%. The values of Glc4 were elevated in PD, especially in patients with infantile presentation.
- In a retrospective review published in 2012, Young et al. [111] determined that urinary Glc4 had high sensitivity (95%) and specificity (84%) as a diagnostic biomarker in a group of pediatric and adult patients evaluated for PD between 2006 and 2011. Glc4 can be elevated in other conditions, so the authors recommended measuring Glc4 in conjunction with GAA activity for diagnostic purposes. They also concluded that Glc4 was useful for monitoring the response to ERT in these patients, especially for patients with infantile PD on ERT. A reduction into or close to the control range during the first few weeks of treatment was considered a good prognostic indicator by the authors. As a complement to Glc4 and other biomarkers, magnetic resonance spectroscopy and imaging techniques are also recommended for assessing the distribution of affected tissues and the degree of PD severity.

Observing the aforementioned results, Glc4 undoubtedly has importance for the evaluation of patients with GSDII. The last obstacle to overcome, for implantation in clinical practice, is related to its accurate measurement in dried urine spot samples and other easily accessible sources, which has depended on the development of robust procedures based on mass spectrometry methods for biological samples [112,113].

### 5.3. Cardiomyopathy in GSDII

#### Human Studies

Myocardial necrosis is the result of a cardiomyopathy that usually occurs due to the excessive storage of glycogen in the heart muscle, producing cardiomegaly. The measurement of TNNT2 as a marker of myocardial necrosis in the absence of acute coronary syndrome [83] is useful for measuring subclinical cardiac pathology in people with GSDII. A study [114] presented a clinical case of a 39-year-old man suffering from PD with no family history of heart disease or other coronary risk factors. He was admitted for emergency. Serum samples showed high values of CK and aspartate transaminase (AST). Echocardiography displayed no abnormalities in the size of the ventricles or their contractibility. However, TNNT2 was positive. The authors consider the presence of TNNT2 to be a very reliable indicator of necrosis of the myocardial muscle.

### 5.4. Skin Fibroblasts as Another Assessment Tissue of GSDII Progression

#### Human Studies

The lysosome-associated membrane protein (LAMP1) is a glycoprotein anchored to lysosomes [115,116]. It was shown that skin fibroblasts from people with PD have levels of LAMP1 up to five times higher than those in skin fibroblasts from control people [117]. Skin fibroblast cell lines from people with PD and healthy people (HP) have been used to investigate glycoprocessing, trafficking, and LAMP1 turnover [118]. It has been shown that the trafficking time for LAMP1 to the trans-Golgi network is 25 min in PD cell lines, compared to 17 min in HP; from the trans-Golgi network to the lysosome, it is 200 min in PD cell lines and 100 min in HP. These observations suggested a delay in glycoprocessing, which the authors suggested may be due to the congestion of the intracellular space from the excessive accumulation of non-digestible material in the lysosomes. In addition, LAMP1 showed a longer half-life in PD cell lines than in HP, which is most likely due to a reduced turnover rate rather than an increase in the synthesis rate. It is not the result of an increase in the glycosylation of this protein since the size of LAMP1 was not significantly different between PD cell lines and HP. Both cell lines (in PD and HP) showed a significant proportion of LAMP1 in the lysosome in its soluble form, with approximately 25% of the total LAMP1 in the lysosome. This soluble form lacked the cytoplasmic tail. The amount of LAMP1 secreted by the cell was 8.5% in HP and 5.6% in PD. The secreted protein could not re-enter the cell, and this secreted form contained the cytoplasmic tail, which indicates that its orientation may not be correct and might explain its secretion. This extended trafficking time for LAMP1 in PD cell lines could be of interest for evaluating progression of the disease.

**Figure 5:**
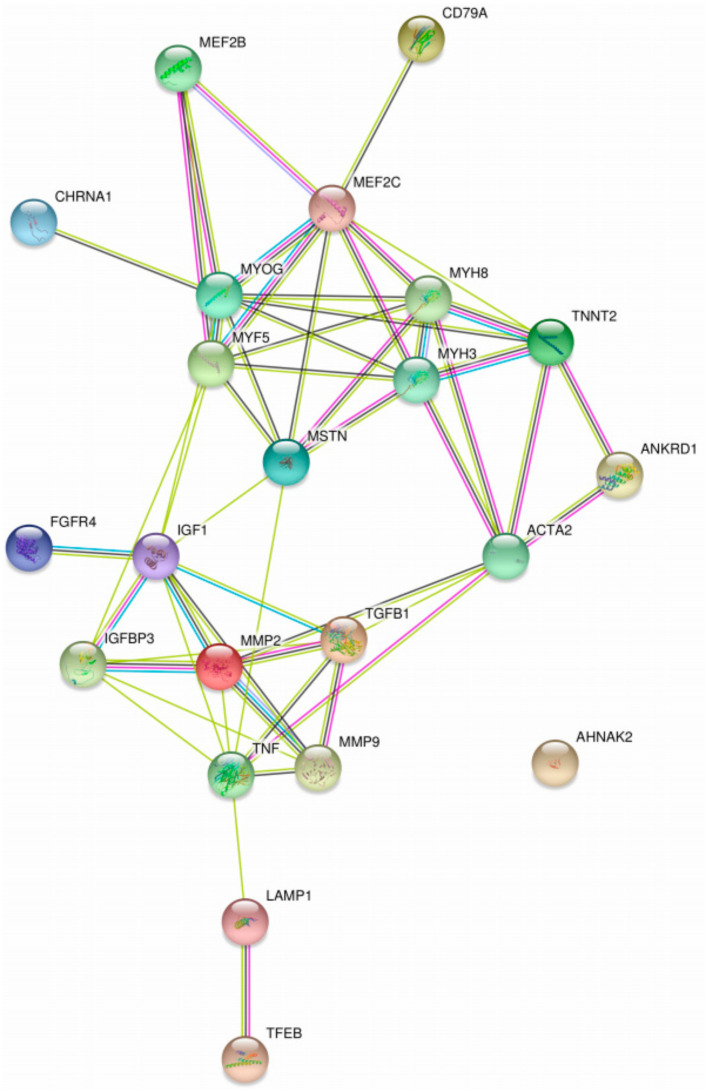
Protein-protein interaction map of possible biomarkers in Pompe disease extracted from Table 3 using STRING.

The STRING database [72] was used to visualize interactions between the biomarkers mentioned in Table 3 (see Figure 5). The central role of proteins such as MSTN (growth/differentiation factor 8), MMP2, IGF1, MYH3, MYH8, MEF2C, and MYF5 in the interaction network influencing GSDII is remarkable.

## 6. Putative Markers in McArdle (GSDV)

### 6.1. Malfunction of Skeletal Muscle in GSDV

#### 6.1.1. Human Studies

As mentioned in background, muscle involvement is common in individuals affected with GSDV and efforts to achieve early biomarkers for diagnosis and prognosis are very welcomed. Santacatterina et al. [119] used reverse phase protein microarrays (RPMA), a high-throughput technique that allows the quantification of a given protein from minute biological samples [120], to quantify and study the putative relevance of 19 proteins as potential biomarkers in a cohort of 73 muscle biopsies (deltoid and quadriceps) including control donors and patients affected by GSDV and other rare neuromuscular diseases. Pyruvate dehydrogenase E1 alpha 1 subunit (PDH E1α), cytochrome c oxidase subunit I (COX1) and human lactate dehydrogenase A (LDH-A) biomarkers were downregulated in patients with GSDV compared to controls. An interesting suggestion by the authors was the β-subunit of the H^+^-ATP synthase/human lactate dehydrogenase A (β-F1-ATPase/LDH-A) ratio being a bioenergetic signature of muscle affectation independent of the different genetic or epigenetic mechanisms involved in the onset of neuromuscular diseases. It was noteworthy that the β-F1-ATPase/LDH-A ratio was elevated in GSDV patients [119].

Selected genes involved in skeletal muscle activity were profiled by Nogales-Gadea et al. [121]. Muscle tissue of 35 patients (aged 17–71 years old) and 7 healthy controls were used in the study. Transcriptomic profiles were compared between 35 McArdle’s disease patients with varying degrees of involvement and seven healthy individuals with sedentary lifestyles as controls. Significant differences (*p* < 0.01) were found in the activity of six genes involved in muscle function: acetyl-CoA carboxylase beta (ACACB), calpain III large subunit (CAPN3), M-cadherin (CADH15), muscle creatine kinase (CKM), muscle glycogen synthase (GYS1), and sarcoplasmic reticulum Ca^2+^ ATPase 1 (SERCA1). Those people affected by McArdle’s disease had fewer transcripts than the healthy controls, i.e., a downregulation independent of the type of mutation affecting the glycogen phosphorylase gene (PYGM), suggesting that the differences in expression patterns are only due to the deficiency of glycogen phosphorylase (GP). In addition, SERCA1 and glycogen synthase (GS) protein levels were decreased in those affected by McArdle’s disease compared to controls by 75% and 50%, respectively. Based on these results, the authors suggested that the absence of GP in McArdle’s patients may result in the downregulation of two control points in the metabolism of skeletal muscles: SERCA1 protein levels and phosphorylation and the amount of GS. The SERCA1 downregulation in patients could impair calcium transport in type II muscle fibers, leading to early onset fatigue.

The proteins most commonly used as plasma markers of skeletal muscle damage are creatine kinase (CK) and myoglobin (Mb) because they are relatively specific for skeletal muscle tissue compared to many other proteins [122,123,124]. Telethonin (Tcap) is another structural protein with expression in skeletal and cardiac muscle [125], so an increase in plasma Tcap after skeletal muscle damage could suggest disruption of the sarcomere and severe damage [126]. Looking for new biomarkers, which would improve early detection of damage in skeletal muscle, Dahlqvist et al. [127] investigated patients affected by Becker muscular dystrophy (BeMD), an X-linked inherited myopathy with a mutation in the dystrophin gene resulting in dysfunctional dystrophin, McArdle disease, and a control group of healthy individuals. Twenty-one subjects affected by either McArdle’s disease, aged 15–62 years, or BeMD were recruited for this pilot study [127] and were subjected to an incremental cycle test until exhaustion. They found that Mb and TNNI2 appeared in the blood soon after induced muscle damage and returned to near pre-damage levels after 24 h. CK had a slower increase and clearing rate, peaking at 24 h after induced muscle damage, and did not return to pre-damage levels after 48 h. An increase in plasma TNNI2 levels was observed only in patients with muscle disease but not in healthy controls, and the concentration of Tcap was not detectable in any of the subjects at any time. Therefore, results showed a faster response and a good specificity of sTnI in comparison with CK and Mb markers. In addition, sTnI’s increase in plasma strongly indicated skeletal muscle damage, so it would be useful to investigate whether sTnI could be used as a marker for treatment response in patients with myositis or other muscle diseases.

Interestingly, Scalco et al. [128] reported that exercise intolerance, myalgia, and rhabdomyolysis (all symptoms shared with GSDV) may be caused by CAV3 mutations. The CAV3 gene encodes caveolin-3, a muscle-specific plasma membrane protein. This is related to several processes involved in the formation of caveolae, which are invaginations of the plasma membrane. The authors found 8 patients with myalgia (*n* = 7), exercise intolerance (*n* = 7) and recurrent rhabdomyolysis (*n* = 2), all typical symptoms of GSDV, with mutations in CAV3. Other features such as hyperCKaemia, reported muscle rippling, percussion-induced rapid muscle contractions, muscle weakness, and hypertrophic cardiomyopathy were either noted at presentation or developed during the study.

#### 6.1.2. Animal Studies

Another study performed by Fiuza-Luces et al. [129] identified specific proteins and pathways involved in endurance-exercise-training adaptations at the muscle tissue level, using a total of 36 male (age: 8 weeks; wild-type: *n* = 18 vs. McArdle’s: *n* = 18) mouse models. The training was successful in inducing a significant improvement in the total running distance of both the McArdle (test *p* = 0.035) and wild-type (*p* = 0.041) mice, but no significant change was found over the same period in the sedentary controls. Mouse body mass showed an increasing trend over time irrespective of genotype or intervention. Proteomic analysis was performed in 10 McArdle mice by quantitative differential analysis via liquid chromatography with tandem mass spectrometry, and protein networks enriched in the differentially expressed proteins were assessed by hypergeometric enrichment analysis. A total of 74 (trained vs. sedentary wild-type mice) and 123 (trained vs. sedentary McArdle mice) differentially expressed proteins were found, respectively. Of these, only three were common to wild-type and McArdle mice: LIM and calponin homology domains-containing protein 1 (LIMCH1), poly (ADP-ribose) polymerase 1 (PARP1), and tigger transposable element derived 4 (TIGD4). It is well known that during exercise, numerous stress signals are transduced to activate intracellular signaling pathways controlling skeletal muscle gene transcription and translation [130,131,132,133]. LIMCH1 and PARP1 could participate in stress pathways controlled by exercise; the absence of LIMCH1 expression impacts the formation of actin stress fibers and the stability of focal adhesions (fundamental structures in the union of muscle fibers ensuring optimal contraction and muscular distention) [134]. The TIGD4 protein belongs to the tigger subfamily of the pogo superfamily of DNA-mediated transposons in humans, but its exact function is unclear. PARP1’s basal activity is crucial for maintaining cellular homeostasis, and its over-activity leads to an increase in protein PARylation, which depletes intracellular NAD+, leading to cell death [135,136]. Endurance-exercise training mediates the expression of PARP1 in both McArdle and wild-type mice but was higher in trained McArdle than trained wild-type mice, as indicated by the high muscle damage. A total of 76 effector proteins were identified and classified into four groups according to their cellular function (14 in adaptive muscle growth, 8 in muscle angiogenesis, 12 in glucose and lipid metabolism and mitochondrial biogenesis, and 48 in remodeling), with four of these belonging to two groups. The small overlap between the groups suggests considerable differences in the physiological adaptations to chronic endurance exercise between the McArdle and wild-type mice. Indeed, the McArdle mice presented a specific and strong expression of mitogen-activated protein kinase 12 (MAPK12) and a potentially substantial number of effector interactions. MAPK12 is one of the four p38 MAPKs that play an important role in the cellular-response cascade induced by extracellular stimuli such as proinflammatory cytokines or physical stress [137]. MAPK12 also plays a role in myoblast differentiation and facilitated glucose transport [138]. It is also related to mitochondrial biogenesis and remodeling according to analyses, with a MAPK12-mediated improvement in mitochondrial function perhaps compensating for the deficit in energy supply owing to glycogen unavailability [129]. Of the proteins, 28% and 22% showed a significant relationship with skeletal muscle adaptations to endurance-exercise training in McArdle and wild-type mice, respectively. In the enriched protein sets involved in muscular adaptations to endurance-exercise training, there were several pathways referring to mitochondria and their functions (mitochondrial structures such as the proton-transporting ATP synthase complex and GO processes such as the respiratory electron transport chain). Conversely, the enriched protein sets within the wild-type groups were related to physiological changes due to focal adhesion, actin cytoskeleton reorganization, and the phosphatidylinositol 3-kinase (PI3K) signaling pathway. McArdle’s most widely represented functions are involved in ATP synthesis, electron-transport-chain processes, the β-oxidation of lipids, and lipid and protein catabolism. The overexpression of PARP1 could be associated with rhabdomyolysis, and PARP1-2 activation is linked to necrosis [136]. The main muscle molecular adaptations are more oriented towards obtaining energy for tissue regeneration in a state of energetic deficit, but wild-type muscle adaptations seem to be more related to supporting tissue maintenance while coping with exercise stress stimuli.

**Figure 6:**
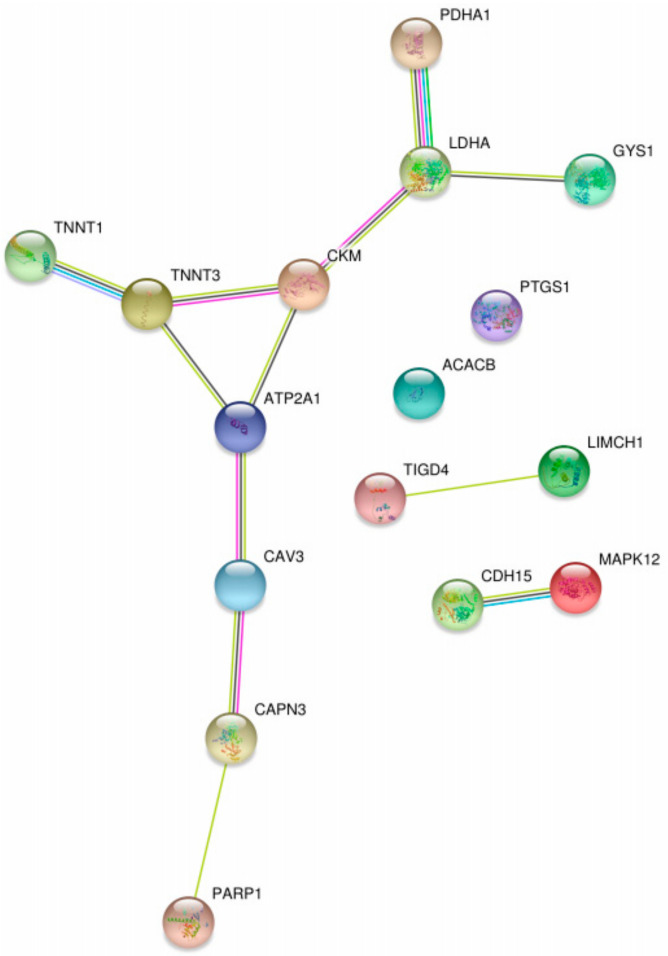
Protein-protein interaction map of possible biomarkers in GSDV extracted from Table 4 using STRING.

The STRING database [72] was used to visualize interactions between the biomarkers mentioned in Table 4 (see Figure 6). The central role of proteins such as CKM, TNNT3 (troponin T3), ATP2A1 (sarcoplasmic/endoplasmic reticulum calcium ATPase 1), and LDHA (Lactate dehydrogenase A) in the interaction network influencing GSDV is remarkable.

## 7. Conclusions

This review is the first summary of possible biomarkers for GSDs reported in the scientific literature; there is no such exhaustive review among the literature consulted. It is important to mention that the reported studies on people affected with any GSD were performed with a very small number of patients.

A very small percentage of new biomarkers have achieved clinical validation (fecal calprotectin and urinary 1,5AG for hepatics GSDs and urinary Glc4 for PD), suggesting that a new important effort must be applied to a broader application of these markers in a clinical environment. Even more, some of these potential biomarkers, once validated, could help gene therapies with checking for undesirable complications in the early stages of therapy. In this scenario, appearance of liver adenomas or carcinomas, bowel inflammation, osteopenia, function of skeletal and cardiac muscle, and other complications derived from GSDs need new biomarkers that will serve a more effective monitorization and treatment than currently validated ones. More studies with a higher sample size are required for a clinical validation of many of the biomarkers presented in this work.

Additionally, there are another group of biomarkers only tested in mice or other animal models. These markers would need to be tested in GSD patients for checking the utility as reliable biomarkers and finally to perform clinical trials. This group has a long way to run in the process for a complete validation.

Increasing information about GSD gene structure, and progress in molecular genetics, have allowed to establish a new standard in the diagnosis of GSDs, improving the accuracy to establish the exact type of GSD and suppressing inconveniences for patients, as previous more invasive methods of diagnosis. However, in the field of prognosis and therapeutics, GSD research is currently centered on the development of gene therapy. In our opinion, this focus on gene editing techniques hides other research lines, such as the search for better biomarkers for improving the quality of life of the patients affected by these pathologies. The biomarkers described in this review might lead us in that direction and open up new horizons for improving diagnosis, prognosis, and therapy.

## Figures and Tables

**Table 1:** List of possible biomarkers in GSDI.

Type	Biomarkers	Sample	Species	Comments	Risks-Complications	Status	References
Diagnosis	HNF1A	Liver	Human	Inactivation in GSDI-related HCA	High risk *	Lack of clinical validation in GSDs	[13,24]
	IL6ST, GNAS	Liver	Human	Mutated in IHCA	High risk *	Lack of clinical validation in GSDs	[13]
	CTNNB1, IL6ST	Liver	Human	Mutated in bHCA	High risk *	Lack of clinical validation in GSDs	[13,15,24]
	IL6ST	Liver	Human	Activation in IHCA	High risk *	Lack of clinical validation in GSDs	[13,14]
	LGR5 and/or GLUL	Liver	Human	High expression in bHCA	High risk *	Lack of clinical validation in GSDs	[13,16]
	PCK1, PCK2, G6PT1, FBP1, GCKR	Liver	Human	Decreased expression in GSD non-tumor livers	High risk *	Lack of clinical validation in GSDs	[13]
	GCK, GPI	Liver	Human	Increased expression in GSD non-tumor livers	High risk *	Lack of clinical validation in GSDs	[13]
	IGF2	Liver	Human	Higher levels from HCA to HCC	High risk *	Lack of clinical validation in GSDs	[24]
	Calprotectin	Faeces	Human	High concentrations in GSDIa-associated IBD	Non-invasive, No risk	Tested in GSDs clinical trials	[41,42,43,44,45]
	SDH, GPDH	Blood (lymphocytes)	Human	Decreased in mitochondrial enzyme activity, resulting in loss of glycolysis	Low risk	Lack of clinical validation in GSDs	[64]
	NADH-H	Blood (lymphocytes)	Human	Increased in mitochondrial enzyme activity, resulting in loss of glycolysis	Low risk	Lack of clinical validation in GSDs	[64]
	HEPC	Liver	Human	Increased in some HCA, mediator in the anemia of GSDIa	High risk *	Lack of clinical validation in GSDs	[26]
	CD11b and CD11a	Blood (neutrophils)	Mouse	Impaired neutrophil migration and adhesion with decreased expression	-	Not yet tested in humans with GSD	[53]
Prognosis	IGF2	Blood	Human	predictor of metabolic control	High risk *	Lack of clinical validation in GSDs	[40]
	miR-32, miR-199a-5p, miR-199b-5p, miR-214, miR-582-5p, miR-130b	Liver	Human	Differential expression in GSDIa HCA	High risk *	Lack of clinical validation in GSDs	[25]
	IL8	Blood	Human	Stimulated by lipids accumulation	High risk *	Lack of clinical validation in GSDs	[18]
	IGF1	Bone	Mouse	Expression promotes osteoblastic activity on bones	-	Lack of clinical validation in GSDs	[54,55,56,57,58,59,62,63,73]
	AMPK, SIRT1, FGF21, β-klotho	Liver	Mouse	Activated in the AAV-NT individuals	-	Not yet tested in humans with GSD	[27,28,37]
Therapeutic	IGF2	Liver	Human	A therapy targeting IGF2 could prevent malignancy	High risk *	Lack of clinical validation in GSDs	[24]
	1,5AG	Blood (neutrophils)	Human	Inhibits the first step of glycolysis and reduces neutrophils survival	Low risk	Tested in GSDs clinical trials	[49]
	miR-224, miR-452	Liver	Human	High overexpression in GSDIa HCA	High risk *	Lack of clinical validation in GSDs	[25]

Abbreviations: inflammatory bowel disease (IBD); hepatocytic nuclear factor 1 alpha (HNF1A); guanine nucleotide binding G protein alpha stimulating (GNAS); hepatocellular carcinoma (HCC); hepatocellular adenoma (HCA); inflammatory HCA (IHCA); β-catenin (CTNNB1); β-catenin activated HCA (bHCA); glycoprotein 130 (gp130 or IL6ST); leucine-rich repeat containing G protein-coupled receptor 5 (LGR5); glutamate-ammonia ligase (GLUL); phosphoenolpyruvate carboxykinase 1 and 2 (PCK1 & PCK2); glucose-6-phosphate transporter 1 (G6PT1); fructose-1,6-bisphosphatase (FBP1); glucokinase regulatory protein (GCKR); glucokinase (GCK); glucose-6-phosphate isomerase (GPI); succinate dehydrogenase (SDH); glycerol-3-phosphate-dehydrogenase (GPDH); NADH-dehydrogenase (NADH-H); biotinidase (BTD); hepcidin (HEPC); interleukin-8 (IL8); insulin-like growth factor type 1 and 2 (IGF1 & IGF2); AMP-activated protein kinase (AMPK); sirtuin-1 (SIRT1); fibroblast growth factor 21 (FGF21); tumor suppressor co-receptor β-klotho; non-tumor-bearing recombinant adeno-associated virus vector-treated GSDIa mice (AAV-NT); 1,5-anhydroglucitol (1,5AG); microRNA (miR); β2 integrin α subunits (CD11a and CD11b). * Biopsies complications (see Appendix A).

**Table 2:** Summary of potential biomarkers in other hepatic GSDs (not in Von gierke disease).

Type	Biomarkers	Sample	Species	Comments	Risks-Complications	Status	References
Diagnosis	Tn	Cardiac tissue	Human	Levels significantly elevated in a GSDIII patient with cardiomyopathy	High risk *	Lack of clinical validation in GSDs	[89]
	Glc4	Urine, Blood (erythrocytes)	Human	Found in GSDIII and GSDIX patients	Non-invasive, no risk/Low risk	Lack of clinical validation in GSDs	[76,77,78,79]
Prognosis	Glc4	Urine, Blood (erythrocytes)	Human	Decreased Glc4 excretions reflected improve in fasting tolerance for GSDIII pediatric patients	Non-invasive, no risk/Low risk	Lack of clinical validation in GSDs	[81]
	Glc4	Urine, Blood (erythrocytes)	Human	Decrease may be secondary to fibrosis progression leading to cirrhosis	Non-invasive, no risk/Low risk	Lack of clinical validation in GSDs	[80]
	IGF1	Blood	Human	Reduced levels may contribute to reduced BMD in GSDIII individuals	Middle risk	Lack of clinical validation in GSDs	[54,55,56,57,58,59,62,63,73]

Abbreviations: insulin-like growth factor type 1 (IGF1); reduced bone mass density (BMD); troponin (Tn); biotinidase (BTD); glucose tetrasaccharide (Glc4). * Biopsies complications (see Appendix A).

**Table 3:** Set of putative biomarkers in GSDII.

Type	Biomarkers	Sample	Species	Comments	Risks-Complications	Status	References
Diagnosis	MYH3, MYH8, MYOG, MYF5, MEF2B, MEF2C, TNNT2, CHRNA1, ANKRD1, AHNAK2	Skeletal muscle	Human	Increased in IOPD	High risk *	Lack of clinical validation in GSDs	[91]
	TGFβ, TNFα, CD79A	Skeletal muscle	Human	Upregulated in IOPD patients	High risk *	Lack of clinical validation in GSDs	[91]
	ACTA2, FGFR4	Skeletal muscle	Human	Increased/unchanged in IOPD biceps, while decreased in IOPD quadriceps	High risk *	Limited value due to variability	[91]
	LacCer	Liver, heart, and skeletal muscle	Human	Increased storage in patients	High risk *	Lack of clinical validation in GSDs	[93]
	TNNT2	Blood	Human	Presence in a clinical case was an indicator of necrosis	High risk *	Lack of clinical validation in GSDs	[114]
	IGF1, IGFBP3	Blood	Human	Elevated in 26 patients	Low risk	Lack of clinical validation in GSDs	[94]
	Glc4	Urine, Blood	Human	High sensitivity (95%) and specificity (84%); very helpful for distinguishing IOPD newborns	Non-invasive, no risk/Low risk	Validated by clinical trials	[82,108,111]
	MMP2, MMP9	Blood	Human	Differential expression with other LSDs	Low risk	Lack of clinical validation in GSDs	[92]
Prognosis	MMP2, MMP9	Blood	Human	Altered protein expression after initiation of ERT in 2 patients	Low risk	Lack of clinical validation in GSDs	[92]
	LAMP1	Skin	Human	Extended trafficking time inside fibroblasts for patients	High risk *	Lack of clinical validation in GSDs	[117,118]
	31^P^ MRS PME	-	Mouse	Elevated in the skeletal muscle of GAA−/− mice	-	Not yet tested in humans with GSD	[95]
	TFEB	Skeletal muscle	Mouse	High expression gave rise to lysosomal clearing of glycogen excess, and improvement of muscle ultrastructure	-	Not yet tested in humans with GSD	[99]
Therapeutic	IGF1, MSTN	Blood	Human	Lower levels in patients before ERT, after ERT is increased	High risk *	Lack of clinical validation in GSDs	[102]
	Glc4	Urine, Blood	Human	Levels correlated with motor response to ERT	Non-invasive, no risk/Low risk	Validated by clinical trials	[103,104,111]
	MMP2, MMP9	Blood	Human	Altered protein expression after initiation of ERT in 2 patients	Low risk	Lack of clinical validation in GSDs	[92]
	31^P^ MRS PME	-	Mouse	High potential for observing metabolic changes after GAA gene therapy	-	Not yet tested in humans with GSD	[95]
	TFEB	Skeletal muscle	Mouse	Treated mice showed glycogen accumulation was almost completely cleared	-	Not yet tested in humans with GSD	[99]

Abbreviations: myosin heavy chain 3 and 8 (MYH3 and MYH8); myogenin (MYOG); myogenic factor 5 (MYF5); myocyte enhancer factor 2B and 2C (MEF2B and MEF2C); cardiac troponin T2 (TNNT2); acetylcholine receptor alpha subunit (CHRNA1); ankyrin repeat domain 1 (ANKRD1); AHNAK nucleoprotein 2 (AHNAK2); infantile-onset Pompe disease (IOPD); transforming growth factor beta 1 (TGFβ); tumor necrosis factor alpha (TNFα); B-cell antigen receptor complex-associated protein alpha chain (CD79A); lactosylceramide (LacCer); insulin-like growth factor type 1 (IGF1); insulin-like growth factor binding protein 3 (IGFBP3); actin alpha 2, smooth muscle, aorta (ACTA2); glucose tetrasaccharide (Glc4); enzyme replacement therapy (ERT); matrix metallopeptidase 2 and 9 (MMP2 and MMP9); lysosome-associated membrane protein (LAMP1); 31^P^ magnetic resonance spectroscopy phosphomonoester (31^P^ MRS PME); acid alpha-glucosidase enzyme (GAA); Myostatin (MSTN); transcription factor EB (TFEB). * Biopsies complications (see Appendix A).

**Table 4:** Experimental biomarkers in GSDV.

Type	Biomarkers	Sample	Species	Comments	Risks-Complications	Status	References
Diagnosis	PDH E1α, COX1, LDH-A, ACACB, CAPN3, CADH15, CKM, GYS1	Skeletal muscle	Human	Downregulated in patients	High risk *	Lack of clinical validation in GSDs	[119,120,121]
	β-F1-ATPase/LDH-A	Skeletal muscle	Human	Upregulated in patients	High risk *	Lack of clinical validation in GSDs	[119]
	SERCA1	Skeletal muscle	Human	Downregulated in patients	High risk *	Lack of clinical validation in GSDs	[121]
	CAV3	Skeletal muscle	Human	Myalgia, exercise intolerance and recurrent rhabdomyolysis appeared in patients with CAV3 mutations	High risk *	Lack of clinical validation in GSDs	[128]
Prognosis	TNNI2	Blood	Human	Increased levels after induced muscle damage, only in patients	Low risk	Lack of clinical validation in GSDs	[127]
	LIMCH1, TIGD4	Skeletal muscle	Mouse	High expression after endurance exercise training in both McArdle and wild-type mice	-	Not yet tested in humans with GSD	[129]
	MAPK12, PARP1	Skeletal muscle	Mouse	Strong expression in McArdle mice after endurance exercise	-	Not yet tested in humans with GSD	[129,137,138]

Abbreviations: pyruvate dehydrogenase E1 alpha 1 subunit (PDH E1α); cytochrome c oxidase subunit I (COX1), human lactate dehydrogenase A (LDH-A); acetyl-CoA carboxylase beta (ACACB); calpain III large subunit (CAPN3), M-cadherin (CADH15); muscle creatine kinase (CKM); muscle glycogen synthase (GYS1); β-subunit of the H^+^-ATP synthase/LDH-A (β-F1-ATPase/LDH-A); sarcoplasmic reticulum Ca^2+^ ATPase 1 (SERCA1); caveolin-3 (CAV3); fast skeletal inhibitory troponin (TNNI2); LIM and calponin homology domains-containing protein 1 (LIMCH1); tigger transposable element derived 4 (TIGD4); mitogen-activated protein kinase 12 (MAPK12); poly (ADP-ribose) polymerase 1 (PARP1). * Biopsies complications (see Appendix A).

## Data Availability

The datasets used and/or analyzed during the present study are available from the corresponding author on reasonable request.

## Appendix A

### Appendix A.1. Complications/Risks Derived from Clinical Interventions

Some of the biomarkers examined in this review are present in different human tissues and their analysis implies organ biopsy, giving rise to a few health risks and complications, mentioned as follows:

### Appendix A.2. Hepatocellular Biopsy

- Common: pain, small intrahepatic or subcapsular hematoma that does not require intervention, hypotension related to a vasovagal response [139,140].
- Uncommon: severe hemorrhage, including the pleural or peritoneal cavity, large hematoma, pneumothorax, subcutaneous emphysema, haemobilia, sepsis and septic shock, puncture of neighboring organs (kidney, pancreas, colon, extrahepatic bile ducts or gallbladder), peritonitis (including biliary), subdiaphragmatic abscess, intrahepatic arteriovenous fistula, rupture of the biopsy needle [139,140].

### Appendix A.3. Skeletal Muscle Biopsy

Complications are minimal, mainly bruising, or wound infections that can be avoided with sterile surgical technique. In young children sedation is necessary, so it is contraindicated in cases where this procedure is required [141,142].

### Appendix A.4. Myocardial Biopsy

The risks are moderate [143,144] and include:

- Blood clots
- Bleeding from the biopsy site
- Cardiac arrhythmias
- Infection
- Recurrent laryngeal nerve injury
- Injury to the vein or artery
- Pneumothorax
- Rupture of the heart (very rare)
- Tricuspid regurgitation

A new, less traumatic, and flexible device is being developed by Grankvist et al. [145]. It allows for safer and more specific sampling of different parts of the myocardium compared to conventional methods.
